# The Efficacy of Double-Heart Nursing in Combination with Seaweed Polysaccharide for Patients with Coronary Heart Disease Complicated with Diabetes: A Pilot, Randomized Clinical Trial

**DOI:** 10.1155/2022/2159660

**Published:** 2022-04-04

**Authors:** Yulei Hu, Yan Wang, Fengwei An, Nini Dai

**Affiliations:** ^1^Department of Cardiology, Jiaozhou Central Hospital of Qingdao, Jiaozhou, China; ^2^Department of Rheumatology, The Fifth People's Hospital of Qingdao, Qingdao, China; ^3^Outpatient Department, The Fifth People's Hospital of Qingdao, Qingdao, China; ^4^Department of Pediatrics, Qingdao Hospital of Traditional Chinese Medicine (Qingdao Hiser Hospital), Qingdao, China

## Abstract

**Objective:**

To study and explore the effect of double-heart nursing combined with seaweed polysaccharide on improving the self-efficacy and quality of life of patients with coronary heart disease and diabetes.

**Methods:**

Eligible 214 patients with coronary heart disease and diabetes who were diagnosed and treated in our hospital between year 2017 and 2020 were randomized at a ratio of 1 : 1 to either control group (seaweed polysaccharide) or observation group (double-heart nursing combined with seaweed polysaccharide). The self-efficacy and quality of life of the two groups of patients after treatment were compared.

**Results:**

The observation group reported a lower blood glucose level after treatment vs. the control group [(6.28 ± 4.49/8.24 ± 2.01) vs. (7.74 ± 4.18/11.41 ± 3.12)] (*p* < 0.05); a lower incidence of lesions in the observation group versus the control group after treatment (*p* < 0.05); and significantly lower SAS and SDS scores of the observation group vs. the control group was observed [(41.27 ± 4.08/43.81 ± 2.93) vs. (62.74 ± 3.48/61.58 ± 3.85)] (*p* < 0.05). Regarding the self-efficacy, the observation group was superior to the control group after treatment (*p* < 0.05). The treatment with double-heart nursing combined with seaweed polysaccharide was associated with the improvement of the quality of life with respect to social function, psychological function, and material life (each *p* < 0.05). The observation obtained a significantly higher satisfaction rate in comparison with the control group [107 (98.13%) vs.95 (88.80%)] (*p* < 0.05).

**Conclusion:**

Seaweed polysaccharide and double-heart nursing might be practical in improving the self-efficacy and quality of life of patients with coronary heart disease and diabetes mellitus, compared with conventional clinical treatment alone.

## 1. Introduction

Coronary heart disease is a common and frequently-occurring disease [1], and it is usually associated with depression and anxiety and other negative emotions, which is not well manageable and controllable [2, 3]. Diabetes is a frequently seen endocrine disease [4] and predisposes to declined immunity. Complications such as diabetic retinopathy, vascular disease, and neuropathy may occur if blood sugar is not favorably controlled. Also, diabetes is a major contributing factor for coronary heart disease. The diabetes combined with coronary heart disease reports more difficulty to control and the dangerousness vs. coronary heart disease alone [5]. In recent years, the morbidity and mortality of coronary heart disease have witnessed a rising trend. As a chronic disease, although the radical cure for coronary heart disease remains unknown, a number of risk factors such as external stimuli have been identified, leading to alternative nursing taken into consideration. Double-heart nursing, a mode adapting to cardiovascular diseases, pays attention to the patient's psychological health, respects the patient's subjective feeling while treating the physical illness, which has been widely applied clinically and achieved remarkable results [6]. Seaweed polysaccharide, a multicomponent mixture [7], has an immunoregulatory role in the physiological function of the human body.

Some studies reported its use as an additive ingredient in food for diabetics. Seaweed polysaccharide has many biological activities, and one of its component of low molecular weight fucoidan sulfate (LMSF) is an inherent intercellular water-soluble polysaccharide in brown algae, mainly composed of fucose and organic sulfate, contains galactose, xylose, and a small amount of uronic acid. The experiments conducted by the Qingdao Ocean University demonstrated that LMSF can directly scavenge peroxyanion free radicals and hydroxyl free radicals *in vitro* and also significantly enhance the SOD binding capacity in serum and tissues *in vivo*. Accordingly, it can serve as a potential drug in lowering blood lipids and preventing atherosclerosis [8, 9]. The present study was designed to explore the treatment effectiveness of double-heart nursing in combination with seaweed polysaccharide on coronary heart disease complicated with diabetes.

## 2. Study Design

### 2.1. Participants

Eligible 214 patients with coronary heart disease and diabetes who were diagnosed and treated in our hospital between year 2017 and 2020 were randomized at a ratio of 1 : 1 to either control group or observation group. In the control group, there were 49 males and 58 females, with an age of 42-81 years and 40-72 years, respectively; in the observation group, there were 55 males and 52 females with an age of 45-76 years and 41-79 years, respectively. It should be noted that there were no significant differences between the two groups regarding the baseline values in the different domains (*p* > 0.05) (See Table 1).

### 2.2. Inclusion and Exclusion Criteria

Inclusion criteria are as follows: in compliance with diagnostic criteria for diabetes and coronary heart disease, patients were informed of the study and voluntarily signed the consent form; this study has been approved by the ethics committee of our hospital.

Exclusion criteria are as follows: allergies to the drugs used in the study; patients with congenital heart disease or psychiatric diseases; and patients who did not complete the treatment due to transfer midway or other reasons.

### 2.3. Interventions

The patients in the control group were given routine symptomatic nursing, including blood glucose measurement, basic nursing, vital signs monitoring, and medication following doctor's orders.

The patients in the observation group were given double-heart nursing combined with seaweed polysaccharide for treatment. Upon admission, routine examination and diagnosis were performed, and then, seaweed polysaccharide was orally administrated.

Double-heart nursing: first of all, emotional counseling nursing should be implemented for patients. Nurses should strengthen active communication with patients, carry out peer education, encourage patients, and guide patients to carry out psychological adjustment. In addition, nurses should inform the family members of patients of the value of social support nursing in detail and guide the family members to accompany and care for patients as much as possible, so as to reduce their psychological burden. Secondly, nurses give health knowledge guidance to patients, inform patients of the pathogenic factors and treatment methods of the disease, and put forward reasonable prognostic suggestions and assist patients in making diet and exercise plans and explain the use methods and precautions of drugs. Nurses help patients strengthen self-management and improve their self-management ability, so as to form good eating habits, medication habits, and exercise habits and fundamentally enhance patients' treatment compliance.

### 2.4. Outcomes

200 ml of blood was drawn intravenously in the fasting state and two hours after meals, and the changes of blood glucose were compared between the two groups.

The adverse lesions including hyperlipidemia, microvascular disease, and eye disease were compared, which diagnoses through laboratory examination and fundus examination.

The self-rating anxiety scale (SAS) and the self-rating depression scale (SDS) were applied to assess and compare the psychological state of the two groups of patients; the score of 55 or below represents normal, the score of 56-65 represents mild anxiety or depression, the score of 66-75 represents moderate anxiety or depression, and the score of 76 or above represents severe anxiety or depression; and lower scores indicate better psychological state.

The purpose-built disease management ability questionnaire was employed to assess self-efficacy of the two groups, with 10-15 suggesting the average disease management ability and 15-20 suggesting the good disease management ability; the higher the score, the better the patient's self-efficacy and disease management ability.

WHO Quality of Life-Abbreviated Version was simplified according to WHO QOL-100 and consists of 26 items in four fields: physical health, psychological function, social relations, and material environment. It mainly evaluates the individual's quality of life. The higher the score, the better the quality of life. Cronbach's of each dimension is *α*. The coefficients are >0.70.

The satisfaction questionnaire developed by our hospital (including medical staff attitude, medical staff efficiency, and medical staff disease explanation) was used to assess the satisfaction and rated as very satisfied, satisfied, not very satisfied, and dissatisfied.

The above indicators were collected at admission and discharge.

### 2.5. Statistical Analysis

GraphPad Prism 8 software and SPSS 22.0 software were employed for graphics plotting and data analysis. Enumeration data [*n* (%)] and measurement data (*x* ± *s*) were verified via *χ*2 tests and *t*-tests, respectively. The level of significance was set at *p* ≤ 0.05.

## 3. Results

### 3.1. Blood Sugar Levels

The observation group reported a lower blood glucose level after treatment vs. the control group [(6.28 ± 4.49/8.24 ± 2.01) vs. (7.74 ± 4.18/11.41 ± 3.12)] (*p* < 0.05), indicating that double-heart nursing combined with seaweed polysaccharide plays a favorable sugar-lowering function in coronary heart disease and diabetes mellitus, as shown in Table 2.

### 3.2. Incidence of Lesions

The chi-square test revealed a lower incidence of lesions in the observation group versus the control group after treatment, suggesting that double-heart nursing combined with seaweed polysaccharide in the treatment of coronary heart disease complicated with diabetes leads to a high safety profile (*p* < 0.05, Table 3).

### 3.3. Mental State

**Figure 1:**
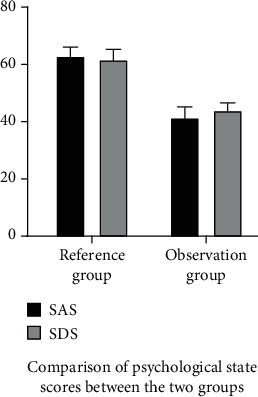
Comparison of psychological state scores between the two groups.

Significantly lower SAS and SDS scores of the observation group vs. the control group were observed [(41.27 ± 4.08/43.81 ± 2.93) vs. (62.74 ± 3.48/61.58 ± 3.85)] (*p* < 0.05, Figure 1). It implies that treatment with double-heart nursing combined with seaweed polysaccharide results in a good mental health after treatment.

### 3.4. Self-Efficacy

Regarding the self-efficacy, the observation group was superior to the control group after treatment (*p* < 0.05, Table 4).

### 3.5. Quality of Life

The *t*-test results showed that treatment with double-heart nursing combined with seaweed polysaccharide was associated with the improvement of the quality of life with respect to social function, psychological function, and material life (each *p* < 0.05, Table 5).

### 3.6. Patient Satisfaction

The results showed that 39 (36.45%) patients in the control group were very satisfied, 56 (52.35%) satisfied, 10 (9.35%) not very satisfied, and 2 (1.85%) dissatisfied; in the observation group, 46 (42.99%) were very satisfied, 59 (55.14%) satisfied, 2 (1.87%) not very satisfied, and 0 (0.00%) dissatisfied. The observation obtained a significantly higher satisfaction rate in comparison with the control group [107 (98.13%) vs. 95 (88.80%)] (*p* < 0.05).

## 4. Discussion

Nowadays, the morbidity and mortality of coronary heart disease continue to rise [10]. However, there remains no radical treatment due to its long course, chronic property and ease to recur, and complications of depression and anxiety [11]. Diabetes is a trigger of coronary heart disease [12], and coronary heart disease combined with diabetes is considered more dangerous and risky than ordinary coronary heart disease [13, 14]. Additionally, issues such as poor compliance and insufficient disease knowledge also exist in some patients [15], thereby necessitating an effective alternative. Dual-heart nursing concentrates on patient's psychological and mental health and subjective feelings and is considered a scientific therapy [16, 17]. Seaweed polysaccharide is a multicomponent mixture and has proven to have a good effect on the treatment of diabetes[18] [19, 20]. According to our results, the observation group outperformed the control group in terms of blood glucose level and the incidence of lesion, probably because sargassum confusum oligosaccharides (SCOs) in seaweed polysaccharides could significantly lower fasting blood glucose levels, protect the structure of liver cells, and increase the abundance of Bacillus and Clostridium XIVa, reduce the abundance of Bacteroides, Allobaculum, and Clostridium IV and play an antidiabetic role through the JNK-IRS1/PI3K signaling pathway [19, 20]. Moreover, our results showed that SAS and SDS scores in the observation group were lower than those of the control group, and the healthy mentality is presumably attributed to that the double-heart nursing was given from the humanistic care while the seaweed polysaccharide was administered. Also, the present study reported a better self-efficacy in the observation group versus the control group. Assumedly, the medical staffs' explanation about the disease knowledge, communication with the patient's family in dual-heart nursing, allows the patient to recognize their symptoms and to manage their emotions, thereby benefiting medication compliance. More importantly, we observed a better quality of life in the observation group, which might be contributed to the administration of seaweed polysaccharide stimulated differentiation and maturation and reproduction of various immune active cells (such as macrophages, T lymphocytes, and B lymphocytes), so that the patient's immune system can be restored and strengthened and further their body function. Double-heart nursing requires the patient to be followed up by the nursing staff after discharge and pays attention to the patient's physical changes and adjusts, which contributes a lot to maintaining good living habits, social function, psychological function, and material life. Notably, treatment with seaweed polysaccharide and double-heart nursing yielded a higher satisfaction in the present study. Consistently, the research conducted by the Yantai Laiyang Central Hospital obtained similar conclusions.

As previously noted, seaweed polysaccharide and double-heart nursing might be practical in improving the self-efficacy and quality of life of patients with coronary heart disease and diabetes mellitus, compared with conventional clinical treatment alone, and it is worthy of wide application and promotion.

## Figures and Tables

**Table 1:** Comparison of general data between the two groups ($\bar{x}\pm s$).

Groups	*n*	Gender	Age (year)	Course of disease (year)	Blood sugar level
Control group	107	49/58	55.01 ± 8.73/54.33 ± 7.95	5.62 ± 4.03	11.65 ± 3.34
Observation group	107	55/52	56.14 ± 6.88/55.72 ± 8.17	5.18 ± 4.67	11.48 ± 3.72
*t*	—	—	1.052/1.261	0.738	0.352
*p*	—	—	0.294/0.209	0.461	0.725

**Table 2:** Comparison of blood glucose levels in the two groups after treatment ($\bar{x}\pm s$, point).

Groups	*n*	FBG	2hBG
Control group	107	7.74 ± 4.18	11.41 ± 3.12
Observation group	107	6.28 ± 4.49	8.24 ± 2.01
*t*	—	2.462	8.835
*p*	—	0.015	<0.001

**Table 3:** Incidence of adverse lesions in the two groups after treatment (%).

Groups	*n*	Hyperlipidemia	Microvascular disease	Eye disease	Total
Control group	107	19	11	8	38 (35.51)
Observation group	107	11	7	5	23 (21.50)
*X* ^2^	—	—	—	—	5.519
*p*	—	—	—	—	0.023

**Table 4:** Comparison of disease management ability between the two groups after treatment ($\bar{x}\pm s$, point).

Groups	*n*	Symptom perception	Disease management	Emotional management	Medication
Control group	107	12.74 ± 3.01	13.71 ± 2.65	12.05 ± 1.58	15.12 ± 2.14
Observation group	107	18.21 ± 1.35	17.87 ± 2.32	17.85 ± 2.65	18.59 ± 1.57
*t*	—	17.152	12.218	19.446	13.524
*p*	—	<0.001	<0.001	<0.001	<0.001

**Table 5:** Comparison of quality of life between the two groups after treatment ($\bar{x}\pm s$, point).

Groups	*n*	Body function	Social function	Mental function	Material life
Control group	107	68.95 ± 2.98	65.85 ± 4.15	64.12 ± 3.58	67.88 ± 2.74
Observation group	107	81.24 ± 3.84	84.15 ± 4.09	83.28 ± 4.65	84.65 ± 3.27
*t*	—	26.115	32.488	33.772	40.661
*p*	—	<0.001	<0.001	<0.001	<0.001

## Data Availability

The datasets used during the present study are available from the corresponding author upon reasonable request.

## References

[1] Tian Y., Deng P., Li B. (2019). Treatment models of cardiac rehabilitation in patients with coronary heart disease and related factors affecting patient compliance. *Reviews in Cardiovascular Medicine*.

[2] Xue Y., Liu G., Geng Q. (2020). Associations of cardiovascular disease and depression with memory related disease: a Chinese national prospective cohort study. *Journal of Affective Disorders*.

[3] Carney R. M., Freedland K. E. (2017). Depression and coronary heart disease. *Nature Reviews. Cardiology*.

[4] Kaul K., Tarr J. M., Ahmad S. I., Kohner E. M., Chibber R. (2012). Introduction to diabetes mellitus. *Advances in Experimental Medicine and Biology*.

[5] Abi Khalil C., al Suwaidi J., Refaat M., Mohammedi K. (2018). Cardiac complications of diabetes. *BioMed Research International*.

[6] Nekouei Z. K., Yousefy A., Doost H. T., Manshaee G., Sadeghei M. (2014). Structural model of psychological risk and protective factors affecting on quality of life in patients with coronary heart disease: a psychocardiology model. *Journal of Research in Medical Sciences : The Official Journal of Isfahan University of Medical Sciences*.

[7] Beaumont M., Tran R., Vera G. (2021). Hydrogel-forming algae polysaccharides: from seaweed to biomedical applications. *Biomacromolecules*.

[8] Luthuli S., Wu S., Cheng Y., Zheng X., Wu M., Tong H. (2019). Therapeutic effects of fucoidan: a review on recent studies. *Marine Drugs*.

[9] Chudasama N. A., Sequeira R. A., Moradiya K., Prasad K. (2021). Seaweed polysaccharide based products and materials: an assessment on their production from a sustainability point of view. *Molecules*.

[10] Dalen J. E., Alpert J. S., Goldberg R. J., Weinstein R. S. (2014). The epidemic of the 20^th^ century: coronary heart disease. *The American Journal of Medicine*.

[11] Wirtz P. H., von Känel R. (2017). Psychological stress, inflammation, and coronary heart disease. *Current Cardiology Reports*.

[12] Bădescu S. V., Tătaru C., Kobylinska L. (2016). The association between diabetes mellitus and depression. *Journal of Medicine and Life*.

[13] Schütt K., Müller-Wieland D., Marx N. (2019). Diabetes mellitus and the heart. *Experimental and Clinical Endocrinology & Diabetes*.

[14] Clodi M., Säly C., Hoppichler F., Resl M., Steinwender C., Eber B. (2016). Diabetes mellitus, coronary artery disease and heart disease. *Wiener Klinische Wochenschrift*.

[15] Meng R., Yu C., Liu N. (2020). Association of depression with all-cause and cardiovascular disease mortality among adults in China. *JAMA Network Open*.

[16] Hu D. Y. (2021). From psycho-cardiology to "five prescriptions for cardiovascular health". *Zhonghua Xin Xue Guan Bing Za Zhi*.

[17] Wei J., Chen X., Wen C. (2021). Analysis of the application of "psycho-cardiology" model in nursing care of acute stroke patients with depression. *American Journal of Translational Research*.

[18] Jiménez-Escrig A., Gómez-Ordóñez E., Rupérez P. (2011). Seaweed as a source of novel nutraceuticals: sulfated polysaccharides and peptides. *Advances in Food and Nutrition Research*.

[19] Cao M., Li Y., Famurewa A. C., Olatunji O. J. (2021). Antidiabetic and nephroprotective effects of polysaccharide extract from the seaweed *Caulerpa racemosa* in high fructose-streptozotocin induced diabetic nephropathy. *Diabetes Metab Syndr Obes*.

[20] Brown E. S., Allsopp P. J., Magee P. J. (2014). Seaweed and human health. *Nutrition Reviews*.

